# Suggested Supports for Improving the Olweus Bullying Prevention Program’s Implementation and Impact in an Under-Resourced Middle School Context

**DOI:** 10.1007/s12310-025-09765-y

**Published:** 2025-05-30

**Authors:** Melissa Washington-Nortey, Terri N. Sullivan, Kevin Sutherland, Rihana Ahmed, Jelani Crosby, Stephani Hitti

**Affiliations:** 1 Department of Psychology, Institute of Psychiatry, Psychology, and Neuroscience, Addison House, Guy’s Campus, King’s College London, London, SE1 1UL UK; 2 Department of Psychology, Virginia Commonwealth University, Richmond, VA USA; 3 Department of Counseling and Special Education, School of Education, Virginia Commonwealth University, Richmond, VA USA; 4 The Faison Center, School and Center for Autism, Richmond, VA USA; 5 Department of Physical Medicine and Rehabilitation, University of Colorado School of Medicine, Aurora, USA

**Keywords:** Olweus Bullying Prevention Program, Bullying, Implementation science, Adolescents

## Abstract

Despite efforts to reduce and prevent incidents, bullying behaviors remain prevalent in schools, leading to poor outcomes for all involved. While the Olweus Bullying Prevention Program is one of the most extensively implemented school environment interventions in the USA, it has yet to yield consistently positive results across contexts, necessitating efforts to understand strategies to bolster its success and that of similar school-wide prevention efforts. This qualitative study used data from focus group discussions with 39 school staff—teachers, administrators and administrative assistants, other non-teaching staff—on the factors associated with implementing the OBPP in their schools. It specifically distilled their perspectives on potential strategies that could further improve the odds of implementation success and impact. Additionally, it examined similarities and differences in these personnel’s perspectives based on their respective roles and responsibilities. The data yielded seven themes, including intensifying training, increasing the efficacy of staff discussions, addressing issues related to time and conflicting priorities intensifying communication, increasing student involvement, increasing parent involvement, and providing staff support to help with implementation. Personnel’s perspectives aligned with their responsibilities in the program, offering insights into the importance of triangulating data from multiple sources while prompting considerations about the school-wide applicability and feasibility of suggestions offered. The potential and feasibility of these thematic suggestions are discussed in the context of the OBPP intervention’s components and provisions and findings from other school-based interventions. Implications for sustainability-based studies of the OBPP and other school-based interventions are also discussed.

## Introduction

Recent statistics show that approximately 20% of students in the USA report being bullied, with teens reporting rates as high as nearly 50% (Schaeffer, 2023). Bullying behavior occurs when an individual experiences ongoing victimization by a person who is perceived to be more powerful (Gaffney et al., 2019; Olweus, 1994; Olweus et al., 2019). Despite the growing concerns about cyberbullying, the school context remains the most common location for victimization (National Bullying Prevention Center, 2020; Schaeffer, 2023; Seldin & Yanez, 2019). US statistics indicate that in contrast to 15% of 12–18-year-olds who report being victims of cyberbullying, 43% say that victimization occurred in school hallways or stairwells, and 42% identified victimization in classrooms (National Bullying Prevention Center, 2020). Victimization also occurs on outdoor school grounds (22%), in bathrooms/locker rooms (12%), and on school buses (8%). The widely reported adverse effects of exposure to bullying behaviors include internalizing behaviors like depression, anxiety, and low self-esteem and externalizing behaviors like substance abuse, truancy, antisocial behavior, and oppositional behavior (Olweus et al., 2019).

Globally, several interventions, policies, and initiatives have been developed and implemented to address bullying behaviors with varied success. Widely used school-based interventions include the Olweus Bullying Prevention Program (OBPP) developed in Norway (Olweus & Limber, 2010a), the KiVA Antibullying Programme by Finnish researchers (Smith, 2019), and the Steps to Respect program developed in the USA (Frey et al., 2005). Although their initial success prompted widescale national and international implementation, subsequent reports suggest that in some contexts, they have not achieved the desired outcomes or sustained impact (Espelage, 2013). In the USA, while medium-to-long-term efforts of programs marked by high levels of fidelity have yielded impactful outcomes in reducing bullying behaviors and resulting victimization (Sullivan et al., 2021b; Olweus et al., 2019; Olweus & Limber, 2010a), programs with lower levels of fidelity have been less successful (Black, 2007; Limber et al., 2004). However, maintaining high fidelity rates, especially in multi-component, school-wide interventions requiring input and involvement from varied contributors, is often challenging. Even at high-fidelity levels, specific contextual differences, such as complexities related to students’ grade levels may impact findings (Farrell et al., 2018, 2021). For instance, students find middle schools in the USA more complex than elementary schools for reasons such as having several subject-specific teachers rather than one dedicated teacher most of the day, as is the case in elementary school. Such complexities, which may hinder the formation of strong teacher-student relationships and make school management more challenging, may also contribute to a higher proportion of positive, significant findings among younger children compared to adolescents (Bauer et al., 2007; Gaffney et al., 2019; Limber et al., 2018; Olweus et al., 2019; Smith, 2016). Consequently, there is a need to better understand supports that increase the odds of successfully implementing and sustaining school-based interventions like the OBPP, particularly among adolescents.

### The Olweus Bullying Prevention Program

The OBPP is one of the most extensively studied anti-bullying intervention programs. Developed by Dan Olweus, it adopts a whole-school approach and aims to establish structures that promote positive interpersonal relationships within schools. The intervention was first implemented in Norway among 5th to 8th graders and resulted in a 50% reduction in bullying behaviors reported after a 2-year implementation period (Olweus & Limber, 2010b). Since then, the intervention has been widely implemented in Norway and many other countries. Components of the intervention include (1) school-level events and initiatives to communicate the school’s stance on bullying behaviors and strategies to address suspected incidents; (2) a Bullying Prevention Coordinating Committee (BPCC), responsible for organizing and overseeing all events and initiatives linked to the program in the school; (3) an individualized component that involves monitoring hotspots noted for bullying incidents and providing on-the-spot intervention to students when potential incidents are identified; and (4) a community component that seeks to involve the community in its activities to further raise awareness and extend the anti-bullying culture beyond a school’s confines (Limber, 2011; Olweus & Limber, 2010a). Thus, successful implementation of the OBPP requires the involvement of (1) teaching and non-teaching staff, like administrators, classroom teachers, librarians, security personnel, cafeteria staff, etc., (2) students, and (3) parents and key community representatives to ensure full coverage of the student body and the school premises, and the inclusion of key community perspectives to promote its success within and beyond the school.

Conceptualizing intervention implementation as a process, the Exploration, Preparation, Implementation, and Sustainment (EPIS: (Aarons et al., 2011) framework has been used to understand the implementation of several school-based interventions, including the OBPP. The EPIS framework outlines a multi-component framework (exploration, adoption/preparation, implementation, and sustainment) to describe implementation in community-based settings such as schools. Notably, the EPIS emphasizes that different implementation strategies, mechanisms, determinants, and outcomes are important for different implementation phases. To illustrate, reporting on the exploration phase, which involves identifying needs and potential solutions, several studies have outlined researcher-led, community-led, and collaborative engagements that have resulted in identifying bullying behaviors as a priority concern and selecting the OBPP as a preferred intervention strategy (Bauer et al., 2007; Black, 2007). The preparatory phase, capturing researcher and school-based preparations ahead of implementation, has often been described in studies reporting implementation findings, which comprise the bulk of OBPP studies. We highlight findings from some of these studies in the subsequent paragraphs.

Findings from the OBPP’s implementation in Europe, the Middle East, and Asia have been largely positive. The first OBPP study in Norway, implemented between 1983 and 1985 among 5th to 8th-grade students, saw significant reductions in students’ rates of being bullied (64%) and bullying others (53%) (Olweus & Limber, 2010b). Subsequent longitudinal studies, including a nationwide initiative involving approximately 500 schools, also yielded substantial significant reductions in reports of bullying behavior among elementary, middle, and high school students, though the high school reports were sometimes weaker in comparison and took longer to emerge (Olweus & Limber, 2010b). In addition, 5-year follow-up investigations of some of these original studies revealed further reductions in reports of bullying behavior (Olweus & Limber, 2010b). In Germany, middle schools that completed an 18-month-long OBPP implementation saw significant reductions in bullying behaviors among victims and perpetrators (Ossa et al., 2021). These effects were stronger among girls and lower middle school students and were retained in assessments completed at 24 months. On the other hand, there were no significant effects in schools that did not complete the OBPP intervention. A teacher-focused effectiveness investigation in Germany also found that while all intervention teachers became more willing to intervene in suspected bullying incidents, the effect was significant only in the OBPP-certified schools and schools that completed the intervention but not in schools that did not complete the intervention as planned (Jantzer et al., 2023). Similarly, a 2008 implementation of the OBPP in Lithuania led to significant reductions in bullying behavior rates among 5th- to 10th-grade girls and 5th- to 8th-grade boys, which were sustained and extended 11 years later (Zuzevičiūtė, 2023). In Iran and Malaysia, the OBPP was linked to improvements in prosocial behavior, reductions in bullying behavior rates among boys with the highest offending rates (Azad & Amiri, 2012), and reductions in varied bullying acts among girls in single-sex schools (Yaakuba et al., 2010). However, limitations such as missing implementation fidelity details, missing statistical evidence, and a limited number of investigations per country and context—as most of these studies, except for those conducted in Norway, represented single-project investigations—preclude conclusions about the OBPP’s impact in these countries.

Outside Norway, the USA is the only other context where the OBPP has been extensively investigated (Gaffney et al., 2019), and these investigations have yielded mixed results. Whereas overall program effects from some studies have shown significant reductions in bullying incidence rates (Limber et al., 2018), others have produced non-significant or less robust results among some sub-groups of students like middle and high school students and students from some racial and ethnic minoritized groups (Bauer et al., 2007; Limber et al., 2018; Olweus et al., 2019). In patterns similar to those identified in some Norwegian studies, many US-based studies sampling both primary and middle school students have identified stronger program effects among elementary school students compared to their middle and high school peers (Limber et al., 2018; Olweus & Limber, 2010b). These findings also mirrored patterns identified in a meta-analysis conducted by Ttofi and Farrington (2011) that included 89 studies from the USA, Norway, Australia, Spain, Canada, Korea, and many others.

Findings from some studies involving middle and high school schools have produced inconsistent or non-significant results or even suggested that the intervention may have increased rates of student bullying incidents. For instance, in an earlier rural South Carolina-based study by Limber and colleagues (Limber et al., 2004) involving two sets of OBPP implementation schools, findings differed across school sets. Only the first set experienced significant and sustained reductions in outcomes, such as victimization and reports of bullying behaviors to parents and school staff (Limber et al., 2004). Stronger effects were observed among boys and shortly after implementation, but the robust effects were rarely sustained at later time points. The OBPP did not yield significant results in the second set of schools. The reduced effects in later years in the first set of schools and the non-significant findings from the second set were attributed to lower levels of implementation fidelity. This was because most of these schools did not adopt the Olweus model and were similar to control schools in terms of the strategies implemented. Later, Black (2007) implemented the OBPP in urban schools in Pennsylvania, USA, and obtained results that further alluded to the importance of fidelity during program implementation. They defined implementation fidelity as the number of core OBPP program components administered as part of the intervention and noted that while broader findings suggested the intervention was associated with increases in bullying incidents, analysis that accounted for implementation fidelity revealed a different pattern. Namely, schools with fidelity rates of 75% or higher evidenced a reduction in bullying incidents, whereas schools with implementation fidelity rates of 75% or lower registered an increase in bullying incidents. As different contributors likely implemented these varied components, the pattern of findings may also indicate a higher probability of success when multiple components and contributors work effectively.

More recently, findings from longitudinal studies have also elucidated the OBPP’s effect on middle school students. Sullivan et al. (2021b) published results from an 8-year implementation of the OBPP in urban middle schools in the Southeastern USA, where significant program effects were observed in several domains, including cyber and relational aggression, as well as physical, verbal, and relational victimization. Although some effects took longer to emerge than others, all were sustained once they surfaced. Yet, there were no significant effects on a measure of school climate. Another longitudinal investigation by Olweus et al., (2019), sampling a wide range of schools and grade levels in Pennsylvania, USA, also achieved significant program-level reductions in all forms of bullying and victimization incidents among elementary, middle, and high school students, which increased in strength over the intervention period. However, sub-group investigations revealed some inconsistencies in grade-level and ethnicity-based findings, with ethnicity-based investigations showing similar patterns for White and Black students in the bullying and victimization domains, but weaker results among Hispanic students.

Taken together, these findings from US middle school students and the international findings from German middle schools highlight the importance of implementation fidelity and allude to potential challenges in achieving high fidelity rates across all intervention schools, across varied domains, and over extended periods, underscoring the need to carefully investigate factors that may be associated with the emergence and sustainment of significant effects. The longitudinal findings from (Olweus et al., 2019) also indicate that investigations like this may be even more impactful in middle schools serving students from minoritized racial and ethnic groups.

As described earlier, the EPIS framework outlines a multi-phased approach to implementing community and school-based interventions, with specific needs, considerations, determinants, and outcomes associated with each phase and driving phasic investigations (Aarons et al., 2011). However, in contrast to the three initial stages, the sustainment phase, which explores questions linked to the factors or circumstances that may have been associated with the OBPP’s outcomes in specific contexts, has received little attention in the literature. Of these, studies identified so far have explored factors, both positive and negative, that have shaped current implementation efforts and their associated findings. These investigations have identified some program, organizational, practical, and contextual factors. At a programmatic level, identified supports include the content of the intervention, its perceived usefulness, the implementors’ familiarity with the intervention, its requirements, and their responsibilities (Coyle, 2008; Sullivan et al., 2021b). Within the school setting, other organizational, practical, and contextual factors like the level of administrative support and staff commitment, the prevailing school culture, the availability of human resources, and the allocation of protected time for implementation have been identified as important (Sullivan et al., 2021b). Additionally, outside the school context, community attitudes and general support for the initiative have also been identified as integral to its success (Coyle, 2008). Although these factors are important, further research is needed to inform the OBPP’s implementation in middle schools if consistent results across schools and different demographics within schools are to be achieved. In the USA, the larger, more impersonal middle school environment that limits students’ consistent interactions with focal classroom-assigned teachers (Davis, 2006), coupled with the increased need for independence, peer acceptance, and identity formation characteristic of the adolescent period (Branje, 2018; Nansel et al., 2003), may affect the intervention’s success. Increased efforts are needed to understand better the context and factors that may bolster impact.

Therefore, this study sought to investigate school personnel’s perspectives on potential supports that could have bolstered the OBPP’s implementation in an under-resourced, urban middle school context to inform future efforts. It also examines similarities and differences in these personnel’s perspectives based on their respective roles and responsibilities to further inform implementation efforts. The manuscript extends beyond reporting on established supports that facilitated implementation to identify and describe additional supports that can improve the current state of findings in the USA. Based on extant literature, we expected to identify suggestions for a more intensive application or calls for the increased availability of previously identified supports such as training and fiscal resources. Yet, we also hoped to discover novel supports and ideas, particularly those that may be more relevant to under-resourced urban middle school contexts with higher proportions of students from minoritized racial and ethnic groups, to bolster the fidelity of interventions in more complex contexts. Investigations into patterns in the suggestions of school personnel based on their respective roles and responsibilities were exploratory.

## Methods

### Sampling and Participants

Participants—teachers, school administrators, and BPCC members—from two urban middle schools in the southeastern USA were sampled using purposive and convenience sampling strategies. We collected data in the summer of 2015. The fall enrollment for 2014 for each school was between 450 and 500 students. Across both schools, most students identified as Black or African-American (61% in School A and 88% in School B). There were also Latine students (32% in School A and 7% in School B), White students (5% in School A and 4% in School B), and students from other racial/ethnic groups (2% in School A and less than 1% in School B). During the 2014–2015 school year, all students were eligible for the federal free school lunch program.

Thirty-nine adults (age range 29 to 75, *Mage* = 51.1; 54% female) participated in the focus groups. Most participants (69%) identified as Black or African-American, 28% were White or European American, and 3% were from other racial/ethnic groups. Separate focus groups were organized for each role in each school (i.e., teachers, administrators and administrative assistants, and BPCC members). Each focus group had approximately six to eight participants. There were eight female and ten male teachers, six female and three male administrators and administrative assistants, and seven female and five male BPCC members, who were teachers or school staff.

### Procedures

Each participant provided written, informed consent before data collection, and the University’s Institutional Review Board approved all study procedures. Data were collected from representatives from each group, who were invited to engage in a focus group meeting held on the school premises. Focus groups were facilitated by either a faculty member or an advanced-level graduate student, who adopted a semi-structured interview approach. Each meeting lasted approximately 2 h. Participants responded to questions about their perceptions of the primary components of the OBPP, the supports and barriers that affected program implementation, and strategies that can facilitate sustainability. Specific questions used in this study are listed in Table 1.

**Table 1:** List of research questions coded in the current study

#	Research question
1	What are barriers that make it more difficult to implement different components of the program?
2	How have school staff addressed barriers associated with implementing different components of the OBPP?
3	What are supports that make it easier to implement different components of the OBPP?
4	What are your suggestions for addressing barriers and further improving different components of the program?

### Data Analytic Process

Data from the interviews were transcribed verbatim, verified by trained research interns, and analyzed using a thematic analysis approach adapted from (Braun & Clarke, 2012). The coding team consisted of one male and four females, comprising a faculty member (*n* = 1), an advanced-level graduate student (*n* = 1), research assistants (*n* = 2), and an undergraduate student (*n* = 1). They identified as Caucasian (*n* = 1), African (*n* = 1), African-American (*n* = 2), and Middle Eastern (*n* = 1). The coding team was trained on open and axial coding procedures (Strauss & Corbin, 1994) in a workshop that lasted approximately 2 h.

Coding occurred in the context of a qualitative study examining the barriers and supports that affected the OBPP’s implementation. Team members familiarized themselves with the verified transcripts and worked in pairs to develop initial codes using open and consensus coding methods. Open coding involved identifying words, phrases, and statements (i.e., quotes) expressing participants’ opinions on potential supports and solutions to addressing implementation challenges shared in response to questions asked during the focus group meetings. For example, one team worked on the question, “What are barriers that make it more difficult to implement different components of the program?” Coding pairs initially worked independently to extract relevant quotes and assign appropriate nodes. Next, the pairs met for consensus open coding, during which they discussed quote-node sets and developed a finalized list of these sets. During the consensus open coding process, coders sometimes identified phrases or statements that appeared to be a better fit for other research questions (e.g., some participants included information about barriers when asked about supports). In such cases, these quotes and their corresponding nodes were placed in an “unrelated but codable spreadsheet” and incorporated under the appropriate consensus coding sheet after other coding team members had reviewed and agreed on its contents. The method ensured that all relevant quotes for each topic or research question were identified during the coding process. Thus, quotes selected for this study were obtained from specific answers to the question, “What are your suggestions for addressing barriers and further improving different components of the program?” and from all other instances where participants shared novel suggestions that were not implemented during the intervention period. Finalized quote-node pairs were subjected to axial coding, which involved using constant comparison methods (Hewitt-Taylor, 2001) to group quote-node pairs by construct similarity to create themes and subthemes. Preliminary themes and subthemes were presented to the coding team, revised based on team feedback, and finalized. The results were also complemented by Table 2, which shows the frequency counts of quotes shared by school staff across the various themes and subthemes.

### Trustworthiness and Positionality

We upheld principles of trustworthiness throughout the process and during data analysis by adhering to essential principles, including credibility, transferability, confirmability, dependability, and the fit of the data within the context (Connelly, 2016; Morse et al., 2002). Strategies used to meet these criteria included (1) prolonged observation and engagement, as the project was longitudinal, (2) researcher sensitivity and responsivity to the needs and desires of participants and the school throughout the research process, (3) updating the school staff on the project’s progress, (4) triangulation of the data collected during data by comparing the results to existing literature, and (5) alignment of methods with research inquiries and data collection procedures (e.g., sampling diverse participants and using mixed methods to assess the project’s impact across multiple levels and domains. See Authors et al., 2018, 2021, 2023).

Additionally, our data collection and coding teams were diverse, representing different racial/ethnic demographic groups, genders, socioeconomic statuses, and levels of training. We view this diversity as a positive factor that helped strengthen our results and reduce biases. However, we recognize that our team was imperfect and that other sources of bias, such as our training protocol or sequence of questioning participants, may have impacted our project. The aforementioned strategies, which increased the variety of data available for triangulation, also helped to mitigate the potential impact of researcher bias on our results. Transparency was enhanced by conducting initial analysis in pairs and subsequently finalizing themes in a group-based exercise to ensure the results were not unduly influenced by specific coders’ perspectives.

## Results

We identified seven themes in the suggestions provided by school staff and parents, including intensifying communication, increasing student involvement, increasing parent involvement, intensifying training, increasing the efficacy of staff discussions, addressing issues related to time and conflicting priorities, and providing staff support to facilitate implementation.

### Intensifying Communication

The first theme, *intensifying communication*, emphasized the need for increased communication across all personnel involved in implementing OBPP. It included two subthemes— *provide updates on student referrals* and *use emails to keep staff updated- that were endorsed by all school staff*. Participants expressed a need for regular updates on the number of reports received and the status of these reports from the Youth Development Specialist (YDS). As one administrator put it, a “*quarterly or monthly review where we sit down and go over the data and review all the information to kind of close the loop or to follow up in those regards and then, if necessary, do some follow-up conditional meetings with parent and student”* would be helpful. They perceived that in the absence of in-person review sessions, the information could be delivered remotely as a report and that consistently providing such updates would provide assurance that cases were being addressed and increase compliance with reporting. The second subtheme highlighted the value of adopting strategies that ensure all members remained abreast with information shared during meetings. A member of the BPCC, who was pleased with the fact that he received email updates explained why by saying, *“I feel that as the only security officer that’s involved which is, I think, a key component… and I can never get here because either I’m doing a lot of things and I’m on call … But one thing I like about it is that um I do get um emails.”*

### Increasing Student Involvement

The second theme, comprised of comments from BPCC members and school staff, focused on increasing student involvement in three main ways, which included three subthemes proposed by BPCC members and school staff. These subthemes emphasized the need to enhance student involvement by *creatively engaging students in programs and activities, making activities more meaningful for students, and establishing a student mentorship component*. A BPCC member stated that it was important to let students know, *“Hey, we want you to participate,”* while others emphasized permitting different creative expressions and involving them in varied aspects of the program, including components like training that are usually reserved for staff. A BPCC member explained the value of collaborating with students by stating, “*Kids will come up with a rap in no time. They will come up with a skit in no time. Some will come up with a poem, it doesn’t take that much. But the fact that they are asked in the beginning, we bring them into an assembly, and we introduce it to them.”* Other participants alluded to the students’ knack for introducing contemporary slang and developing slogans that would contextualize the program and increase its relevance for their generation. Beyond advocating for intentional, creative engagement from the program’s inception, teachers also felt that it was essential to ensure these activities were meaningful, engaging, rewarding, relevant, sufficiently varied, and implemented using diverse formats to prevent boredom. For instance, one teacher shared *that “switching in different activities with the journal writings or the technology”* would maintain student involvement because, on some occasions, students.

“would say, ‘we did this yesterday’...‘we already, we already talked about this’ and so if there could be like some type of, um, you know, transition or-or something to kinda take the discussion to another level or you know, I don’t know something, something that can, you know, if it is going to be a-a repetition or a review of what they did from the day before, um, I don’t know, something, something to help to keep that conversation going.

Several participants also believed that establishing a student mentorship program across grade levels was another way to secure student involvement and interest in the OBPP. One BPCC member described this by saying, “*if we started a nice mentoring program between eighth-graders and sixth graders, or even seventh and eighth-graders with sixth graders, have a little program WITHIN… where the students are talking to each other.”* They asserted that such a program could capitalize on the awe that lower middle school students have for their older peers to promote a more communal atmosphere of shared responsibility, encouraging popular peers to engage in prosocial behaviors instead of antisocial behavior. However, one participant felt that while such mentorship programs could be beneficial, they would be more effective if they involved high school students, as there was a greater deference to and admiration of these older students.

### Increasing Parent Involvement

Like the points on increasing student involvement, participants believed that increasing parent involvement by actively engaging parents in various aspects of the program, organizing fun events tailored to specific parent interests, and addressing logistical challenges would enhance the project’s impact. Participants from all groups described the need to actively educate parents because, as one administrator explained, *“the root of much of the bullying comes from social media. And so we need to let parents know that, you know, to nip situations like this, we need them to help do that at home. By monitoring their behavior and things that they’re doing on the internet. And their phones.”* An administrator also suggested involving parents in the reinstatement process after any type of suspension such that instead of having parents simply sign off on their children’s behavior contracts, they would “*have a conversation with the parent, …remind them of the consequences, and then what will follow should this behavior repeat itself. … [For instance,] the second time, you know, you [the parent] need to be a part of a meeting, a training, or something.”* Other suggested methods of involving parents included inviting them to the school kick-off events and requiring them to complete assignments and arbitration meetings with their children. Participants also noted that planning events of interest to parents, such as those featuring performances by their children, would increase parent engagement. But as one teacher shared,

“It’s not just inviting parents to come, [but] inviting the whole family to just come because, you know sometimes the parents cannot leave their kids behind at home, they don’t have a babysitter, they don’t want to spend money on [a] babysitter. So basically, it could be a family thing, you know, they could bring their children over or family over or anybody they can trust.”

They also recommended taking active steps to eliminate obstacles that might hinder participation. For instance, proactively anticipating the challenge of getting information out to parents, one teacher stated that it was critical for school authorities to *“check their email address if they have one, so we can communicate to them, [via] email. Hopefully, with Spanish translation as well. Especially if, you know if, uh especially when emailing or sending letters to them. Get their address and email address. So we can [engage in] such communications*.” The rest recommended providing transportation to and from and food at events, experimenting with long-term rewards-based incentives, and providing child care.

### Intensifying OBPP Training

In addition to intensifying communication and increasing student and parent involvement, participants also believed that improving knowledge about the OBPP components could be achieved by *increasing and intensifying school staff training, organizing refresher training sessions, and conducting small-scale training sessions*. Administrators and BPCC members drew attention to the fact that, in some cases, knowledge gaps in what constituted bullying behavior could result in a child not receiving timely assistance. As one administrator discussed, the YDS “*may end up with a referral in his door, and as he talks, he realizes that it’s not bullying. And so we lose sight of the kids who ARE being bullied because you are spending a lot of time, uh, having discussions about things that really are not bullying.”* Consequently, there were calls for “*everybody [to be] involved in the process—staff, parents, community workers, everybody” and for systems to be instituted that ensured new or substitute staff were trained and aware of their expectations.*

Administrators and BPCC members also expressed a desire for booster training sessions to supplement the initial training sessions. One BPCC member stated,* “if we had refresher talks, let’s say, during staff meetings, I think that would help. Just like we do when we have our team meetings*.” Specifically, they believed that monthly sessions led by the research team or sessions that incorporated illustrative videos could highlight challenging implementation issues and enhance staff competency. A few also believed that the average size and composition of training groups were important to consider. For instance, a BPCC member recommended including students in small-group booster training sessions to address any limitations in understanding what constitutes bullying behavior. He explained this by stating,

“A lot of our students start off with an idea, thinking they know what bullying is, but a lot of times when you, like, directly address them about like, “hey! You, you know you’re bullying this kid right now!” they do not want to believe it. And they, we don’t have the time to take that step back. And a lot of the training, like, when you talk to an adult, they’re going to remember like, the procedures in like five minutes, no problem. Having the kids in there [the training sessions] maybe, to do some of that role-playing with us, again, a quarter of the way, halfway through the year, I think, we can retrain everybody.”

### Increasing the Efficacy of Staff Discussions

Another area where participants felt improvements could be related to the efficacy of staff discussions: periodic meetings held by staff at the different grade levels to discuss updates related to implementation and dissemination (Limber et al., 2004). Only BPCC members contributed to this theme and its subthemes. They suggested improving the discussions by *identifying the appropriate leader to facilitate these meetings, protecting meeting times on the school’s master calendar, and creating dynamic agendas to guide discussions*. BPCC members indicated that identifying the appropriate leader for these meetings was critical because,

“If someone, you know, that doesn’t instill [or generate] a lot of conversation, can’t facilitate that type of conversation is trying to lead that meeting, it’s going to just not go anywhere and will be over in 5 min, but if it’s, you know if it’s someone that’s open to facilitating a discussion and concerned about it...it could work.”

The BPCC members indicated that not having a protected time for staff discussions negatively impacted these meetings because other scheduled meetings took precedence over staff discussions. Moreover, even when held, school staff sometimes drifted into talking about other topics unrelated to OBPP. To address such challenges, one BPCC member outlined a potential solution, saying, “*If you forgo one of your planning periods and one of our weekly team meetings … and we hold one of those meetings, as we’re not talking about, you know, anything else today, we’re talking about students and possible bullying situations and behaviors.”* Participants were also convinced that it was important to “*have a very detailed agenda and have training on how to facilitate those meetings”* to make them more efficient and effective.

### Addressing Issues Related to Time and Conflicting Priorities

Administrators and BPCC members also suggested addressing issues related to time and conflicting priorities more generally. They described having several responsibilities that made it challenging to attend OBPP-related meetings and events or complete the paperwork needed to report potential situations involving bullying behaviors. One administrator suggested that to address this challenge*, “it would have to be something that starts the planning process now [at the beginning of the term] to interweave it to some degree in the school calendar.”* A BPCC member also outlined how different OBPP-related activities could be scheduled on other days to help ensure participation by saying, *“If it’s possible, the school may designate certain days for certain after-school meetings. You know like Mondays could be maybe uh anti-bullying meetings, where Tuesdays could be the full-staff meetings, or where Wednesdays could be departmental.”* Another BPCC member added that it might even be beneficial to* “look at the district-wide calendar also as far as meeting schedules.”*

### Providing Staff Support to Help with Implementation

The final theme addressed suggestions related to the provision of support staff, sometimes by the research team and a concerted effort at the school-level, to help with implementation. Administrators and BPCC members asserted that the higher incidence of conflicts, particularly at the beginning of the school year, necessitated having permanent and additional staff available and poised to manage incidents. A BPCC member said, *“In the beginning of the year…it’s going to take a village, it’s not going to just take the bullying prevention specialist, it’s going to take all of us. You know, and it does, you know, fall on all of us.”* Also alluding to the need for consistent support, given that the YDS split time between schools, an administrator expressed that,

“It would be nice if the intervention specialist or youth specialist was here on a daily basis. That would uh, help expedite the process even quicker. And I think we would be able to get more timely, more timely actions, even though there’s an alternate that helps here, but the expertise of the specialist, um, goes further.”

A summary of the themes and subthemes are displayed in Table 2 below.

**Table 2:** Themes, subthemes, and counts

Themes/subthemes	Participants		
	Admin	BPCC	School staff
*Theme 1: Intensify communication* Includes suggestions about how communication can be increased to ensure that all personnel are updated on issues related to student referrals and meeting updates	2	3	1
Subtheme 1.1: Provide updates on student referrals—Refers to participants’ expressed desire to receive updates on referrals made about specific students	2	2	1
Subtheme 1.2: Use emails to keep staff updated	0	0	1
*Theme 2: Increase student involvement* Includes suggestions about strategies to increase student involvement in and uptake of the program	0	6	7
Subtheme 2. 1: Creatively engage students in programs and activities—Refers to participants’ desire for students to be involved in different activities and programs	0	5	2
Subtheme 2.2: Make activities more meaningful for students—Includes comments expressing a desire for all activities to be developmentally appropriate and to have real-world significance	0	3	5
Subtheme 2.3: Establish student mentorship component—Includes suggestions for a mentoring component to be included to engage older students more purposefully	0	1	1
*Theme 3: Increase parent involvement* Includes suggestions about increasing parent engagement to facilitate a more significant impact	7	10	4
Subtheme 3.1: Actively involve parents in different aspects of the program—Refers to participants’ beliefs that it was essential to include parent education as a critical component of the program and implement strategies to ensure their active involvement in different aspects of the program	6	6	1
Subtheme 3.2: Organize fun events based on parents’ specific interests—Refers to participants’ beliefs that organizing fun events that incorporate activities valued by parents would increase parent involvement	3	3	3
Subtheme 3.3: Address logistical challenges—Refers to suggestions for addressing common challenges that hinder parent participation and involvement in meetings and events	5	3	2
*Themes 4: Intensify training* Includes suggestions to strengthen program implementation by organizing small-scale booster training sessions, intensifying training on specific aspects of the program, and ensuring that all new teachers are trained	3	7	0
Subtheme 4.1: Increase and intensify school staff training—Includes suggestions highlighting a need to adequately train all school staff (i.e., old and new) on the different components of the OBPP	3	3	0
Subtheme 4.2: Organize refresher training sessions—Includes calls for booster sessions to be organized throughout the year as a means of maintaining implementation	1	2	0
Subtheme 4.3: Organize small-scale training sessions—Includes suggestions to hold smaller-scale, brief grade-level training sessions with students instead of long school-level training initiatives with staff only	2	0	0
*Theme 5: Enhance the efficacy of staff discussions* Includes suggestions to increase the efficacy of staff discussion meetings by designating skilled teachers as leaders, protecting meeting times, and creating detailed agendas to guide each meeting	0	5	0
Subtheme 5.1: Designate skilled teachers as leaders—Refers to suggestions about the qualities a leader of staff discussion groups must possess	0	1	0
Subtheme 5.2: Ensure staff discussion periods are protected on the school’s master schedule—Includes calls for meetings like staff discussion to be included on the school’s master calendar to ensure consistency	0	2	0
Subtheme 5.3. Create dynamic agendas to guide staff discussions—Includes suggestions about increasing staff discussions’ effectiveness	0	3	0
*Theme 6: Address issues related to time and conflicting priorities* Includes suggestions to prioritize OBPP activities to ensure that they are implemented as expected	1	5	0
*Theme 7: Provide support staff to help with implementation* Includes suggestions for the research team to hire full-time support staff like youth development specialists who can help with addressing issues potentially related to bullying behaviors immediately after they occur	3	1	0

## Discussion

In light of the inconsistent findings associated with the OBPP’s implementation among adolescents, particularly in the USA, we aimed to investigate potential supports that could strengthen implementation efforts, enhance the program’s impact among students and school staff, and facilitate its sustainment in under-resourced school settings. Thematically analyzed interview data from teachers, BPCC members, and administrators yielded seven main themes for improvement that included *intensifying communication, increasing student involvement, increasing parent involvement, intensifying training, increasing the efficacy of staff discussions, addressing issues related to time and conflicting priorities,* and *providing staff support to help with implementation.* As anticipated, some themes replicate findings from the extant OBPP literature while highlighting the need for greater emphasis during implementation. Others represent novel contributions to the field that may be particularly beneficial for implementation in similarly under-resourced contexts.

### Perspectives on Potential Supports to Boost the OBPP’s Implementation in Under-Resourced, Urban Middle School Contexts

The themes mirroring factors identified in the extant literature as OBPP implementation supports included *addressing issues related to time and conflicting priorities* and *providing staff support* like the YDS to assist with implementation. These suggestions allude to some of the most frequently reported challenges associated with implementing the OBPP and other evidence-based interventions (Naylor et al., 2015; Sullivan et al., 2021b). Yet, they are particularly relevant to under-resourced urban middle schools like those involved in this study, where staff turnover is high, and teachers report having heavy workloads and high levels of stress (Albright et al., 2017; Bower, 2013; Simon & Johnson, 2015). Even where dedicated staff like a YDS and school counselors exist, they may be assigned additional responsibilities that may detract from their counseling and prevention responsibilities (Kim & Lambie, 2018). Strong organizational leadership support is a key driver of sustained implementation (Aarons et al., 2011; Naylor et al., 2015; Sullivan et al., 2021a, 2021b). Schools with strong administrator support and endorsement of the program prioritize the OBPP’s activities and often restructure their operations to ensure implementation (Coyle, 2008; Sullivan et al., 2021a, 2021b). However, in challenging work contexts such as these, where demands on and turnover among administrative leadership and staff may be high, structures like protected time and periodic training, instituted to ensure continued implementation may be disrupted. Even previously endorsed programs may be terminated by new leadership. Recent directives from the US federal government requiring schools to terminate all Diversity, Equity, and Inclusion programs or risk losing federal funding (Associated Press, 2025) illustrate how a change in leadership can result in changes to the programs and interventions offered in schools. Therefore, additional strategies are needed to ensure continued implementation. For example, schools can partner with relevant community-based organizations or creatively source funds and additional resources from the community to sustain critical interventions.

These themes also represent important inner context factors—factors prevailing within a context that can either facilitate or hinder an intervention’s delivery (Aarons et al., 2011) and should be considered during the exploratory and preparatory phases of the intervention’s implementation. For instance, interventionists can investigate the extent to which suggestions outlined in the manuscript prevail within their contexts and collaborate with their teams to devise appropriate mitigation strategies. Theory of change maps (De Silva et al., 2014) can also support such exercises by outlining the desired goals and important milestones that pre-empt their achievement. These factors must also be monitored during the implementation and sustainment phases to ensure that all concerns are mitigated and that the intervention’s outcomes are not negatively impacted. Careful monitoring may also reveal other innovative factors and characteristics that can bolster implementation efforts.

More novel themes not explicitly described in the literature on supports impacting the OBPP’s implementation include *intensifying communication, increasing the efficacy of staff discussions, intensifying training*, and *increasing student and parent involvement*. The need to intensify communication becomes even more salient when considering the holistic structure of the OBPP. Successful implementation of the OBPP requires support and investment from all teaching and non-teaching staff and effective communication of strategies to staff, students, parents, and the community (Olweus & Limber, 2010b). Consistent with other studies (e.g., (Unnever & Cornell, 2004), some staff in the current study mentioned instances when they did not receive consistent updates after submitting reports or felt reports had not been addressed promptly, which reduced their enthusiasm for the intervention. Others were in positions that hindered their ability to receive consistent updates or reports from students and staff, which could impact the intervention’s success (Washington-Nortey et al., 2024). There are data suggesting that providing staff and students with clear evidence of change/impact and instituting monitoring systems to check compliance can increase adherence. For instance, Magnusson et al., (2011) found that teachers were more likely to implement a physical activity intervention when they noticed improvements in their students’ productivity levels. Similarly, staff may have felt that increasing communication about the intervention’s activities and sharing regular updates about reports and their resolution strategies with teachers, non-teaching staff, students, and the community could increase adherence and buy-in from all involved. The recommendations to improve staff discussions allude to the quality of a specific system developed to facilitate communication and increase staff competence and confidence regarding the OBPP. Staff discussions aim to stimulate the school’s organizational structures around the OBPP, are typically led by trained staff, last approximately 90 min, and should be held consistently bi-weekly or monthly (Limber et al., 2004). However, in practice, it can be challenging to coordinate and organize consistent, effective meetings, given the competing demands of the middle school schedule and the continual responsibilities of school staff during the day (Limber et al., 2004). Staff’s perceptions of these meetings can also impact the consistency of attendance as, Naylor et al. (2015) found when examining engagement with other evidence-based interventions. Therefore, while these meetings may not be the ideal forum for extensive training-based discussions, identifying skilled leaders and providing well-structured outlines to guide the discussions, as suggested by participants, may increase the quality, usefulness, and diversity of topics discussed.

In addition, the participants requested more intense and consistent training. This also aligns with findings from other evidence-based interventions (Magnusson et al., 2011) which also provides some insight into why participants may have perceived a need for additional training beyond the standard OBPP training. For instance, Cunningham et al. (2016) reported that school staff found it challenging to detect bullying behaviors because the acts did not always align with their perceptions of what constitutes bullying behaviors and because the acts were not confined to a single context. Similarly, Sullivan et al. 2021b noted that teachers’ difficulties in distinguishing between bullying behaviors and conflicts could hinder their ability to consistently detect bullying behaviors. These examples illustrate three nuanced training needs: teachers’ perceptions, the multi-contextual nature of behaviors, and the challenges of differentiating these behaviors from conflict. Several studies highlight effective training and trainees’ perceptions of the intervention materials and requirements as critical factors influencing the successful implementation of interventions (Magnusson et al., 2011; Naylor et al., 2015; Salmon et al., (2011). When trainees perceive the intervention and its components as difficult to understand and implement, they are less likely to implement the intervention effectively.

Another suggestion offered was to include students in the training and planning components of the OBPP, thereby providing students with a greater opportunity to shape the intervention’s implementation and become familiar with all essential components. Student-led initiatives and interventions can transform a student body and the overall school climate. For example, when students in the United Kingdom and four European countries—Greece, Spain, Romania, and Slovenia—participated in an initiative to implement small-scale student-led sustainability projects in their communities, they realized that their actions altered their teachers’ perceptions of them, helping them earn greater respect and attention. Their teachers, particularly those with an authoritarian bent, also became more willing to delegate responsibilities to students (Vare, 2021). Perhaps the students’ actions may have revealed aspects of their personalities that engendered higher levels of trust and confidence, as Washington-Nortey et al. (2024) found in a study where teachers and students collaborated on diverse academic and non-academic projects. Such strategies may also align better with the development needs and interests of adolescents, given their desire for greater autonomy, involvement, and ownership of their lives and the phenomena that interest them.

Lastly, participants felt that increasing parental involvement in the OBPP would increase its impact. Although increasing parental involvement has been identified as a potential support, this can be challenging in practice. Black (2007) found this to be one of the most challenging components of the OBPP to implement, particularly when there was a history of dissatisfaction or tension between parents and the school or specific school staff. Similarly, another OBPP study (Coyle, 2008) found that parents’ resistance to inclusive values (i.e., welcoming new residents into their community) was a major limiting factor in promoting anti-bullying efforts in the school, even when students were receptive to the program. Practical challenges such as parents’ busy schedules, lack of awareness about existing school programs or initiatives, communication and language difficulties in migrant communities, and any negative perceptions about school staff’s willingness to engage or collaborate with parents may also hinder parent involvement (Hong, 2009; Sullivan et al. 2021b). However, in line with our participants’ recommendations to actively include parents in activities Wassell and Hawrylak (2021), found that a Spanish school’s open-door culture and intentional efforts made parents feel welcome and increased their participation in events and unsolicited visits to the school. Interestingly, this school also organized activities that directly benefited parents, including adult English classes and cultural programs to learn about their diverse home cultures. These acts may have communicated value and respect to the parents, making them more willing to participate in other activities indirectly related to them. Black (2007) also found that targeted outreach to parents through existing community networks and partnerships with key parent advocates were beneficial. Additionally, the knowledge gained by organizing such activities and outreaches may further school staff’s knowledge about their parent population and help achieve two additional recommendations shared by our participants: planning other relevant activities and addressing unique logistical challenges facing parents and the broader community, a widely reported barrier to participation (Hong, 2009; Author, 2021).

### Differences in Perspectives Shared Across Participating Groups

In line with recommended thematic analysis approaches, the values of themes and subthemes included in this manuscript were not based on their frequency or proportional representation in the data (Braun & Clarke, 2021). Instead, themes and subthemes were developed based on their unique and nuanced contributions to understanding our topic of interest. However, complementing our analysis with a table showcasing thematic representations across the groups analyzed facilitated the observation of group-based suggestion patterns worth noting. Specifically, although the density of contributions differed across groups, some themes comprised comments from all participating groups. Others were composed of contributions from specific participating groups only.

The themes featuring contributions from all participating groups (i.e., teachers, administrators, and BPCC members), precisely the motions to intensify communication and increase parental involvement, may point to key implementation components affecting intervention delivery at all levels and by all individuals. Such themes may also expose critical contextual challenges, which, if addressed, could aid the intervention’s delivery and ensure its success. For example, several studies reveal that achieving high levels of parental involvement in under-resourced schools located in equally challenging community contexts is often difficult (Hong, 2009). Yet, success among parents can result in cultural shifts within and outside the intervention school (Bal, 2018; Washington-Nortey et al., 2024).

Having oversight responsibilities for an intervention’s implementation may provide a vantage point for identifying key programmatic, socio-cultural, and socio-political factors affecting its success (Garan, 2022; Tintoré et al., 2022). This may explain why themes related to addressing time constraints and conflicting priorities, employing additional staff, and increasing training were raised exclusively by administrators and BPCC members, who had specific administrative responsibilities for implementing the OBPP. Even effective programs can increase workloads, as staff need to learn new skills for the intervention’s implementation (Chitiyo & Wheeler, 2009). Among teachers in stressful, volatile contexts with high workloads, learning and implementing new strategies may be even more challenging (Larson et al., 2018). The administrators may have noticed the added strain placed on staff by their OBPP-related responsibilities, prompting their calls for protected implementation time and additional support. Yet, our findings also show that administrators, including BPCC members, may play a vital role in identifying these and other programmatic needs that can streamline implementation efforts. The absence of contributions from school staff may be due to several factors. They may have perceived the original amount of training associated with the intervention as adequate or may have possessed other competencies relevant to their employment roles, which may have reduced their perceived need for additional training. Alternatively, they may have refrained from suggesting options that might increase program-related requirements, given their workloads, or they may have had limited capacity to identify programmatic needs, given their more specialized roles in the intervention.

Although all staff with administrative responsibilities may identify programmatic needs necessitating further support or intervention, there may be specific needs that are apparent only to personnel exclusively tasked with administering the intervention. In our study, proposals to strengthen staff discussions, an OBPP-specific initiative, were made exclusively by BPCC members. The more detailed understanding of the intervention required to oversee its delivery within their school’s context may have provided insight into the value of each intervention component and its effect on the intervention’s overall success. Staff without this intimate knowledge may be unable to identify excluded or inefficiently implemented components, their potential impact on the intervention’s success, or the value of strengthening those components.

Lastly, the calls for greater student involvement, shared exclusively by BPCC members and school staff but not general administrators, are also insightful. As a reminder, BPCC members included teachers and non-teaching staff, who regularly interfaced with students. Theirs and other school staff suggestions may reflect insights obtained from their consistent interactions with students, as teachers are noted to intentionally and persistently engage students to acquire extensive knowledge about their skills and experiences, which ensures effectiveness during teaching (Mayer & Marland, 1997).

### Implications for School Policy and Practice

Several school policy and practice recommendations can be gleaned from this study. Suggestions to address conflicting priorities and frequent staff changes allude to the need for (1) regular internal evaluations and reviews to establish the intervention’s value and sustain its implementation in lieu of less effective interventions and other competing interests, (2) the integration of the intervention’s activities with the school curricula to allocate protected time for the intervention’s consistent implementation, and (3) the implementation of strategies to retain core implementation staff and reduce the potential for interruptions linked to frequent staff changes. Aaron and colleagues (2011) recommend the use of internal reviews to establish the impact of programs and justify their continuation. Borrowing strategies from the business sector where customer reviews have been shown to sustain investment in products (Dwidienawati et al., 2020; Huang, 2018), internal reviews structured to the school’s specifications, may also convince new administrators and staff of their value, increasing their willingness to adopt and implement the initiatives. Alternatively, integrating intervention activities into school curricula and programs can help establish these programs as core tenets of the school’s culture, mandating their implementation regardless of administrative or staff changes, which may help ensure sustainability. Where possible, school boards with longer tenures of service could be made responsible for reviewing and encouraging the implementation of such programs in less stable environmental contexts. Schools may also consider adopting strategies to preserve a critical mass of trained staff or partnering with research teams to ensure that new staff are adequately trained and adequate oversight is available. Doing so may also safeguard implementation fidelity over time as staff are less likely to be overwhelmed by many responsibilities and can develop expertise in identifying and managing incidents in their schools.

The communication-based suggestions call for more effective approaches to improving communication within, to, and across staff. All staff members must be regularly updated on the school’s efforts and in team-specific forums. Strategies such as bilingual messaging and community-based meetings outside the school environment can empower community members and increase their level of investment in the program. Increasing opportunities for more informal interactions between teachers and students can also help nurture positive student–teacher relationships and allow staff to earn their students’ trust, resulting in an increase in student reports to staff (Washington-Nortey et al., 2024). Considering these factors and strategies during the preparatory and implementation phases of the intervention project can increase their chances of success and ensure intervention sustainment over extended periods.

Additionally, the nuanced training needs and requests for greater inclusion may also be worth further consideration. Booster training sessions that incorporate practical, contextualized examples based on staff and student experiences may be more relevant for specific contexts, and including students in these trainings may increase inter-student and staff accountability. The broader inclusion of students in other program components may also lead to the adoption of more student-friendly activities and the establishment of a subculture around the intervention, further increasing their investment, participation, and potential sustainment. Yet, in the process, interventionists must guard against introducing strategies that deviate from the core principles or aims of the program, as this can increase the risk of intervention failure (Hall et al., 2016).

Lastly, the differences across participant groups, which reflect their specific roles in delivering the intervention, warrant consideration when adopting these suggestions. Interventionists should consider the extent to which these proposals apply to the broader school context or whether they should be applied to specific sub-groups within and outside the school. This will increase efficiency by ensuring that personnel are not unduly burdened and that resources are allocated appropriately during the intervention’s delivery.

### Limitations and Future Directions

There are a number of limitations to consider. First, although the perspectives from varied school staff responsible for different aspects of the OBPP intervention enriched the study, adding the perspectives of students, parents, and researchers would have introduced dimensions that we did not capture. This is especially important given the suggestion for greater student involvement shared by participants. Including student voices may draw attention to experiences more salient to the student body and reveal gaps in the administration and delivery components that adults may have overlooked. Parental and community input may also shed light on essential family and community factors impacting the intervention’s success within and outside the school. However, these parents and community members must be involved in the formative and implementation stages of the program and empowered to provide informed feedback on all aspects of its implementation. Researchers’ views may also provide insight into factors that influence the research-school collaboration and how these can be harnessed to strengthen implementation efforts.

Second, although the suggestions outlined seem plausible and consistent with the literature on implementation factors affecting evidence-based interventions in schools, their effect on the OBPP remains largely untested. Therefore, we cannot guarantee their success in under-resourced contexts, such as our target schools or other school contexts within and outside the USA. Before their adoption, schools implementing the OBPP are also encouraged to consider the extent to which these strategies can be feasibly implemented in their contexts. Researchers may also consider conducting context-specific Delphi studies with school staff based on these findings to identify factors that should be prioritized in their schools when implementing the OBPP or similar interventions.

Third, beyond the feasibility and generalizability of our findings, it is also important to highlight that while this study advances the body of work investigating factors associated with sustaining interventions by collating suggestions that were not explored during the intervention period, it does not propose a framework for sustainably implementing the OBPP in under-resourced contexts. A framework of novel and time-tested strategies identified during implementation may advance the field by strengthening OBPP implementation efforts in under-resourced contexts, resulting in more consistent findings across contexts.

In conclusion, this study examined school staff’s perspectives on strategies to enhance the OBPP’s success and impact within their school’s context. Based on the shared data, we identified seven themes that provide clear suggestions for improved implementation, many of which represent novel contributions to the OBPP literature. Implementing these suggestions may help ensure the program’s success in under-resourced contexts and provide insight into strategies to implement other holistic school-based programs in the USA and elsewhere.
